# Supplementary material for: Detection of M2-Type Anti-Mitochondrial Autoantibodies against Specific Subunits in the Diagnosis of Primary Biliary Cholangitis in Patients with Discordant Results
Source: Diagnostics (Basel). 2023 May 24;13(11):1840. doi: 10.3390/diagnostics13111840 (PMC10252559; doi:10.3390/diagnostics13111840)
Supplement: Supplementary file 1 [file diagnostics-13-01840-s001.zip › diagnostics-2333848-supplementary.pdf]

Supplementary Table S1. Immunological and clinical data of all studied patients

Group/ Patient	AMA-M2 IFA (titer)	ELISA (U/mL)	DB-E2nonSep		DB-E2sep				ALP	Biopsy	Diagnosis	Comments
			3E2	nAg (mainly PDC-E2)	nPDC	PDC-E2	BCOADC-E2	OGDC-E2				
A/1	Negative	22.8	73+++	2 o	-	-	-	-	Elevated	Yes (compatible with PBC)	PBC	Probable systemic lupus erythematosus
A/2	Negative	57	24+	1 o	-	+	-	-	Normal	No	Not PBC criteria (Metabolic associated fatty liver disease)	
A/3	Negative	44	29+	1 o	-	+	-	-	Elevated	Yes (compatible with PBC)	PBC	Elevated GGT/cirrhosis
A/4	Negative	46.2	36++	3 o	-	+	-	-	Normal	Yes (compatible with AIH)	AIH	
A/5	Negative	26.8	47++	10 (+)	-	+	-	-	Normal	Yes (compatible with AIH)	AIH	Cirrhosis
A/6	Negative	66.1	38++	2 o	-	-	++	-	Normal	No	Not PBC criteria (Alcoholic and MAFLD cirrhosis)	
A/7	Negative	77.4	45++	7 o	-	-	++	-	Normal	Yes (compatible with AIH)	AIH	
A/8	Negative	70.4	29+	2 o	-	-	++	-	Elevated	No (due to patient's comorbidities)	Not PBC criteria	
A/9	Negative	58.5	40++	4 o	-	-	+	-	Normal	Yes (compatible with AIH)	AIH	
A/10	Negative	78.6	66++	3 o	-	-	++	-	Elevated	No	PBC	Positive anti-gp210 autoantibodies
A/11	Negative	104.9	40++	4 o	-	-	++	-	Elevated	No (lost to follow-up)	Probable PBC (lost to follow-up)	
A/12	Negative	116.9	38++	2 o	-	-	++	-	Elevated	Yes (compatible with PBC and AIH)	PBC+AIH	
A/13	Negative	41.1	38++	2 o	+	+	+	-	Elevated	No (patient's refusal)	Not PBC criteria (Unclear)	Elevated GGT and poor response to ursodeoxycholic acid
A/14	Negative	71.2	40++	2 o	+	+	+	-	Normal	No	Not PBC criteria	
A/15	Negative	62.1	99+++	18+	+	-	-	+	Normal	No	Not PBC criteria	
A/16	Doubtful AMA-M2 pattern 1/40	71.4	51++	5 o	-	-	+++	-	Elevated	No	PBC	Positive anti-Sp100 autoantibodies

A/17	Doubtful AMA-M2 pattern 1/80	89.6	94+++	6 o	-	-	+++	-	Normal	No (not follow-up and died)	Not PBC criteria	ANCA-MPO vasculitis
A/18	Doubtful AMA-M2 pattern 1/80	97	146+++	3 o	-	-	+++	-	Elevated	Yes (compatible with PBC)	PBC	
A/19	Doubtful AMA-M2 pattern 1/80	70.4	60++	4 o	+++	-	-	+++	Elevated	Yes (compatible with PBC)	PBC	
A/20	1/40	23.4	21+	12 (+)	+	-	+	-	Elevated	Yes (compatible with PBC)	PBC	
A/21	1/40	56.2	39++	4 o	-	-	+	-	Normal	No	Not PBC criteria	Systemic lupus erythematosus
A/22	1/160	98.4	40++	1 o	-	-	++	-	Non assessable due to clinical context	No (lost to follow-up)	Not PBC criteria (Alcoholic cirrhosis)	
A/23	1/160	97.7	74+++	11 (+)	-	-	+++	-	Normal	No	Not PBC criteria	
A/24	1/160	96.1	90+++	13 (+)	+	-	+++	-	Normal	No (patient's refusal)	Not PBC criteria	Elevated GGT and positive anti-Sp100 autoantibodies
B/1	1/320	145.1	221+++	193+++	+++	+++	+++	++	Elevated	Yes (compatible with PBC and AIH)	PBC + AIH	
B/2	1/320	162	226+++	211+++	+++	+++	+++	+++	Elevated	No	PBC	
B/3	1/320	113.1	191+++	196+++	+++	+++	++	-	Elevated	Yes (compatible with PBC)	PBC	
B/4	1/320	110.8	107+++	89+++	++	++	++	-	Normal	No	Not PBC criteria	
B/5	1/320	116.7	213+++	219+++	+++	+++	+++	-	Normal	Yes (severe steatosis)	Not PBC criteria	Positive anti-Sp100 autoantibodies
B/6	1/320	159.8	153+++	163+++	++	+++	+++	-	Normal	Yes (compatible with PBC)	PBC	Elevated GGT
B/7	1/320	157.8	208+++	185+++	++	+++	+++	-	Elevated	Yes (compatible con PBC)	PBC	
B/8	1/320	100.8	156+++	173+++	+++	+++	-	++	Normal	No	Not PBC criteria	
B/9	1/640	113.8	220+++	228+++	+++	+++	+++	-	Elevated	Yes (compatible with PBC and probable AIH)	PBC + AIH	
B/10	1/640	116.7	209+++	213+++	+++	+++	-	-	Elevated	Yes (compatible with PBC and AIH)	PBC + AIH	

DB-E2nonSep: DB with non-separated three E2 subunits; DB-E2sep: DB with separated three E2 subunits; o: negative; (+) (6-10): doubtful; + (11-25): weak positive; ++ (26-50): moderate positive; +++ (>50): positive; ALP: Alkaline Phosphatase; PBC: Primary biliary cholangitis; AIH: Autoimmune hepatitis; MAFLD: Metabolic dysfunction-associated fatty liver disease.
